# Scientific Machine Learning for Elastic and Acoustic Wave Propagation: Neural Operator and Physics-Guided Neural Network

**DOI:** 10.3390/s25123588

**Published:** 2025-06-06

**Authors:** Nafisa Mehtaj, Sourav Banerjee

**Affiliations:** Integrated Material Assessment and Predictive Simulation Laboratory (iMAPS), Department of Mechanical Engineering, Molinaroli College of Engineering and Computing, University of South Carolina, Columbia, SC 29201, USA; nmehtaj@email.sc.edu

**Keywords:** scientific machine learning, wave propagation, physics-guided neural network, neural operator, deep operator network, Fourier neural operator

## Abstract

Scientific machine learning (SciML) offers an emerging alternative to the traditional modeling approaches for wave propagation. These physics-based models rely on computationally demanding numerical techniques. However, SciML extends artificial neural network-based wave models with the capability of learning wave physics. Contrary to the physics-intensive methods, particularly physics-informed neural networks (PINNs) presented earlier, this study presents data-driven frameworks of physics-guided neural networks (PgNNs) and neural operators (NOs). Unlike PINNs and PgNNs, which focus on specific PDEs with respective boundary conditions, NOs solve a family of PDEs and hold the potential to easily solve different boundary conditions. Hence, NOs provide a more generalized SciML approach. NOs extend neural networks to map between functions rather than vectors, enhancing their applicability. This review highlights the potential of NOs in wave propagation modeling, aiming to advance wave-based structural health monitoring (SHM). Through comparative analysis of existing NO algorithms applied across different engineering fields, this study demonstrates how NOs improve generalization, accelerate inference, and enhance scalability for practical wave modeling scenarios. Lastly, this article identifies current limitations and suggests promising directions for future research on NO-based methods within computational wave mechanics.

## 1. Introduction

Monitoring structural performance for damage and durability is instrumental in different engineering disciplines, including aerospace [1,2], civil [3], mechanical [4], and naval [5,6,7] structures. Structural health monitoring (SHM) now provides automated, real-time insights, moving beyond the limitations of traditional non-destructive testing and evaluation (NDT&E) [8,9]. An SHM system consists of an in-service data collection setup and signal analysis capabilities. The core concept is to collect structural responses with distributed sensors, followed by analyzing them to extract damage-sensitive features and predict the health status using a physics-based or data-driven model [10,11]. Over the past decades, a broad range of SHM techniques have been introduced for practical applications [12]. Among them, guided wave-based SHM techniques are widely adopted in the community [13,14,15].

Guided waves are specific types of elastic waves in ultrasonic and acoustic frequencies that propagate in solid plates or layers, governed by the structural form or geometric boundary of the medium [16,17]. Therefore, the propagation characteristics depend on the density and elastic properties of the medium. Two key features make guided waves highly effective for damage detection: short wavelengths at high frequencies and low attenuation over distance. Due to this nature, they are highly sensitive to small defects and efficient at covering large structural areas [12]. While guided waves operate over larger structural scales, surface acoustic waves (SAWs) are suited for surface-sensitive or micro-scale SHM applications. Beyond their diverse applications in biosensing [18], microfluidics [19], and MEMS [20], SAWs are extensively used for non-destructive material characterization [21,22]. Their ability to detect minute surface disturbances makes them well-suited for localized damage monitoring [20]. Thus, it is evident that the success of building robust and reliable SHM critically depends on the study of elastic and acoustic wave analysis. Given this necessity, efficient numerical tools are indispensable to facilitate the study of wave analysis.

To this date, there are many numerical methods available for wave analysis. Among them, finite element (FE)-based methods are most common [23,24]. To name a few more: spectral element method (SEM) [25,26,27], finite difference method (FDM) [28], boundary element method (BEM) [29], mass spring lattice model (MSLM) [30], finite strip method (FSM) [31,32], peri-elastodynamic [33], cellular automata [34,35], elastodynamic finite integration technique (EFIT) [36], etc. These techniques are well-established and have been effectively serving for decades as standard practice. However, there are some key concerns with these methods. These techniques are computationally very demanding due to their mesh-based nature. Thus, solving higher-dimensional problems using these methods becomes challenging. This particular issue is addressed as the curse of dimensionality (CoD) [37]. Secondly, if the grid size is not sufficiently small relative to the wavelength, discretization errors arise, noticeably compromising the resolution [38]. Also, the Gibbs phenomenon is another well-known numerical artifact characterized by spurious oscillations near non-smooth or discontinuous regions. This issue is common in most computational methods due to the reliance on polynomials, piecewise polynomials, and other basis functions [39,40,41]. Despite their individual advantages and disadvantages, all methods share the common challenge of high computational cost [42].

As an effort to lessen the computational expense, researchers proposed many semi-analytical methods. The distributed point source method (DPSM) is one of the most used techniques. It uses displacement and stress Green’s function in its meshless semi-analytical problem formulation. This method is comparatively more accurate and faster, especially in the frequency domain, than FEM, BEM, and SEM [37,43]. However, this model tends to match the required conditions at some specific points (apex) only, which makes the simulated wavefield a bit weaker than expected [38]. The local interaction simulation approach (LISA) is another noteworthy time domain semi-analytical method [39,40,41]. This technique is computationally heavier as it requires additional local interaction of material points in temporal and spatial domains. Parallel computing is a must to efficiently simulate this method. However, the issue of computational burden persists instead of further progress, making the existing numerical and semi-analytical models impractical for real-world applications [42].

In recent years, there has been a notable shift toward leveraging machine learning (ML) techniques for wave propagation modeling [11,44]. ML methods have the exceptional ability to capture high-level features. Their ability to capture the relation between multidimensional data and target variables is incredible [45]. Moreover, these methods work as an effective solution to the issue of computational expenses associated with existing physics-based models. However, this fact does not lessen the importance of these physics-based models, as they play a key role by providing the ground truth to train the ML models. With the growing data from these physics models and the breakthroughs of ML, the term “scientific machine learning (SciML)” has come to the forefront in many areas. SciML especially refers to the inclusion of domain knowledge (physical principles, constraints, correlations in space and time, etc.) in the ML models through data or modification of the architecture [46,47,48,49,50]. The advantages of SciML include (1) a meshless solution technique, thus no issue of CoD; (2) performs better in higher-dimensional space with advanced neural network (NN) architecture [51,52]; (3) gradient-based optimization instead of linear solvers [53,54]; (4) nonlinear representation of NNs and no reliance on linear piecewise polynomials, (5) offers solutions for forward and inverse problems under the same optimization problem [55,56,57].

Owing to its proven benefits, the field is witnessing diversified research efforts. Thus, the current literature of SciML refers to different nomenclature such as “physics-guided”, “physics-enabled”, “physics-based”, “physics-informed”, “physics-constrained”, and “theory-guided”, to name a few. Faroughi et al. [58] classified the SciML models into four prime methods: physics-guided neural networks (PgNNs), physics-informed neural networks (PINNs), physics-encoded neural networks (PeNNs), and neural operators (NOs). Among these four models, PINNs and PeNNs are considered physics intensive. On the other hand, NOs and PgNN approaches are mostly data driven.

**Figure 1:**
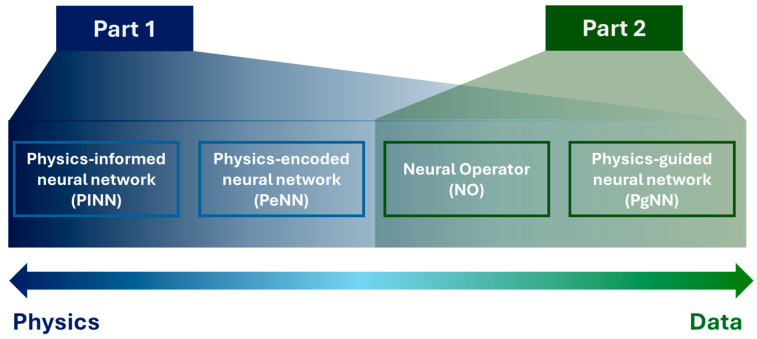
Outline of the two-fold study for the application of physics-driven artificial intelligence tailored for wave propagation.

This article is a direct continuation of Ref. [59], which reviewed the progression of the four SciML approaches and their definition in the context of wave propagation. The first part of this article thoroughly discusses the underlying physics of acoustic, elastic, and guided waves. The latter part of this study concentrates on only the physics-intensive PINN model and its application in different engineering fields involving wave propagation. Figure 1 outlines the topics addressed in this two-part review. In this article, the authors focus on data-driven approaches, as mentioned in Part 2 in Figure 1. Based on the definition, the PgNN is the oldest data-driven method to learn patterns from wavefields with and without damage. Based on the literature, the number of articles combining deep learning (DL) and SHM has surpassed the previous records every year until now [60]. In addition to guided wave signals, researchers in this field utilized vibration signals, images, acoustic emission signals, etc., to train the off-the-shelf statistical methods. The records show that, among different data types, vibration signals (displacement, acceleration, strain) are the most used ones for the PgNN approach. In the context of different DL methods, the convolutional neural network (CNN) is the most adopted one for damage-based feature extraction. A good number of review papers already exist covering PgNNs for guided wave-based SHM [61,62,63,64,65]. Thus, this article particularly emphasizes the newly emerging neural operators (NOs).

The structure of this article is as follows. Section 2 presents the underlying algorithms of neural operators (NOs) applied to wave propagation problems. Section 3 reviews the use of various NO frameworks in modeling acoustic, elastic, and guided wave phenomena. Section 4 concludes this paper by summarizing key findings, highlighting current challenges, and suggesting directions for future research. For the theoretical formulation of wave equations and related physics, readers are referred to Part 1 of this study [59].

## 2. Data-Intensive SciML Models: Architecture and Algorithms

SciML models are primarily employed for three core purposes: (i) solving PDEs (PDE solver), (ii) discovering governing equations from data (PDE discovery), and (iii) learning solution operators (operator learning). PDE solvers include methods such as PINNs [66], PeNNs [67,68,69], and PgNNs [70]. These models can be highly data-dependent or physics-dependent based on the objective and problem type (inverse or forward). These approaches are mostly useful for solving existing PDEs for a specific set of physical constraints and fixed parameters for parametric PDEs. However, the second category regarding PDE discovery works on the data to reveal the structure or coefficients of the PDE without any prior knowledge of the equation. Sparse identification of nonlinear dynamical systems (SINDy) by Brunton et al. [71] is an excellent example of this powerful tool.

In particular, this section focuses on the 3rd one, operator learning. It is a purely data-driven approach to solve a family of PDEs, both parametric PDEs and nonparametric PDEs. To this date, operator learning has primarily focused on forward problems, aiming to generalize the solution space [72]. Before diving into different neural operator architectures, a brief description of operator learning in the context of wave equations has been presented first in Section 2.1. Later, different neural operator architectures already utilized to simulate wave propagation are discussed.

### 2.1. Concept of Operator Learning

A series of established studies [72,73,74] on universal approximation theorems demonstrate that sufficiently large shallow (two-layer) NNs can approximate any continuous function within a bounded domain. This theoretical guarantee makes NNs a powerful approximator. Thus, scholars extended this capability of NNs to approximate operators in functional maps from one function space to another, unlike the usual vector-to-vector mapping. To be more specific, operator learning is a data-driven framework designed to approximate nonlinear operators that map between infinite-dimensional Banach or Hilbert spaces of functions [75,76]. The origin of this concept can be traced back to early work in regression on function spaces, later formalized through rigorous approximation theory for neural operators [77]. According to a study by Kovachki et al. [78], neural operators are currently the only class of models proven to support both universal approximation theory and discretization invariance, unlike conventional deep learning models that rely on fixed-grid inputs and fail to generalize when those grids are reformed.

**Figure 2:**
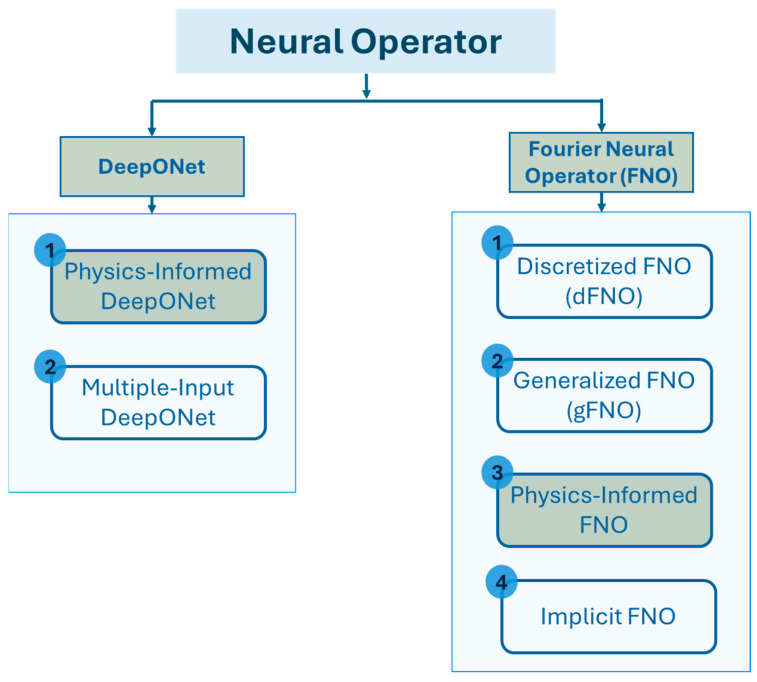
Variants of DeepONet and FNO architectures [85]; models highlighted in green have been utilized for wave equation modeling.

**Figure 3:**
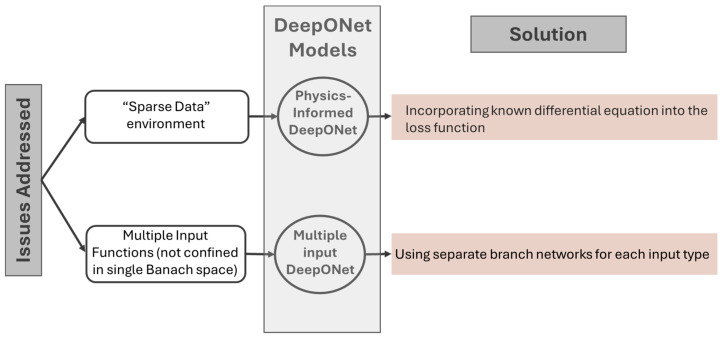
Motivation and architectural adaptations in DeepONet.

**Figure 4:**
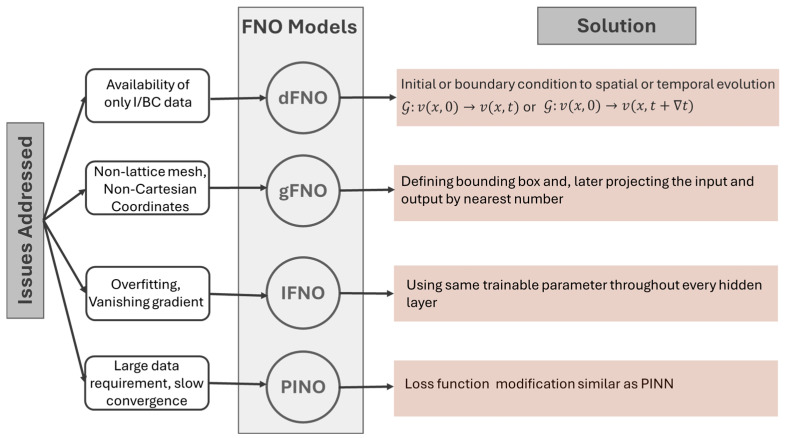
Motivation and architectural adaptations in FNOs.

Based on this concept, until now, scholars in this field have proposed various neural operator architectures, namely deep operator network (DeepONet) [79], Fourier neural operator (FNO) [80], wavelet neural operator (WNO) [81], Laplace neural operator (LNO) [82], convolutional neural operator (CNO) [83], spectral neural operator (SNO) [84], and many more. To date, only DeepONet and FNO, along with some of their variants, have been applied to wave PDEs. Figure 2, based on Goswami et al. [85], illustrates some of the major DeepONet and FNO variants, highlighting those used in wave propagation studies. Figure 3 and Figure 4 summarize the key challenges addressed by each variant and outline the corresponding solution strategies within the two neural operator frameworks.

*Problem Formulation*: To illustrate the concept of neural operators in the context of wave equations, this paper considers the homogeneous 3D wave equation expressed by Equation (1).

(1) $$\left(\frac{\partial^{2}u}{\partial x^{2}}+\frac{\partial^{2}u}{\partial y^{2}}+\frac{\partial^{2}u}{\partial z^{2}}\right)=\frac{1}{c^{2}}\frac{\partial^{2}u}{\partial t^{2}}$$

Here, u(x,y,z,t) denotes the wave displacement field, (x,y,z) the spatial coordinates, and t the temporal variable. To keep the problem simple, homogeneous Dirichlet boundary conditions are considered. Considering space-dependent wave speed c(x,y,z) in the material, Equation (1) can be rewritten in the following form:

(2) $$\partial_{t}^{2}u(x,t)-c^{2}(x)\nabla^{2}u(x,t)=0,\;\forall (x,t)\in \Omega \times (0,T)$$

(3) $$\left.\begin{array}{c} u\left(x,0\right)=u_{0}\left(x\right),\forall x\in \Omega \\ \partial_{t}u(x,0)=v_{0}(x),\forall x\in \Omega \end{array}\right\}$$

(4) u(x,t)=0,∀(x,t)∈∂Ω×(0,T)

Here, x = (x,y,z) is used as a vector notation for the 3D space. The material system is modeled within the spatial domain $\Omega \subset R^{3}$, where the wave speed is defined by a bounded function c(x) within Ω. The initial condition of the system is presented by Equation (3), where $u_{0}(x)$ denotes the initial displacement and $v_{0}(x)$ denotes the initial velocity. The boundary condition for the system is presented by Equation (4). Here, the final time T>0.

The primary goal here is not to find the solution of the PDE explicitly every time, but to learn the operator. In this context, frameworks such as DeepONet and FNOs aim to learn the operator $Q:f\mapsto u_{\theta }(x,t)$, where Q maps the input function *f*, which consists of initial conditions and the medium properties (for parametric PDEs) or only initial conditions (for nonparametric PDEs with fixed parameters), to predict the solution $u_{\theta }(x,t)$. Here, θ refers to the trainable weight w, and bias b are parameters. Q can be expressed in the integral form as follows:

(5) $$Q\left(f\right)=u_{\theta }(x,t)=\int_{\Omega }\;G(x;\xi ,\;t\;;\tau )u_{0}(\xi )d\xi +\int_{\Omega }\;\frac{\partial G}{\partial \tau }(x;\xi ,\;t\;;\tau )v_{o}(\xi )d\xi$$

More generally,

(6) $$Q\left(f\right)\left(x,t\right)=\int_{\Omega }\;K(x;\xi ,t;\tau )f(\xi ,\tau )d\xi$$

Here, in Equation (5), G(x,t;ξ,τ) is Green’s function for the system where it represents the fundamental solution describing the response at point x and time t due to a unit impulse applied at location ξ and time τ, subject to the same boundary and initial conditions as the original wave equation. In Equation (6), the K(x;ξ, t ;τ) is the generalized kernel function from the formulation perspective of a neural operator, which will be thoroughly discussed in later sections. In the context of wave propagation, parametric and nonparametric PDEs are discussed herein to understand the following sections better. If the velocity profile c(x) on the entire structure is fixed (i.e., nonparameterized), which could be inhomogeneous and anisotropic, then the operator Q that is intended to be learned is parameter-independent. However, if the intention is to find the mapping operator Q that can map any given velocity profile c(x) to an output displacement wavefield u(x,t), then the operator Q is a parameter-dependent neural operator.

For wave propagation problems, it is necessary to find the entire displacement wavefield u(x,t) in a material or structure (i.e., at every spatial point (x,y,z)) over a period (0−T). Let us divide this time period T into two segments, which we can call *train* and *predict* segments as $T^{train}\;\in \left(t_{0}-t_{1}\right)$ and $T^{predict}\;\in \left(t_{2}-T\right)$. To clarify further, it is to be noted that for the parameter-independent neural operator Q, the c(x) profile is not provided as input, as it is irrelevant for a fixed wave speed profile. Please note that the fixed wave speed profile does not mean a constant homogeneous speed over the entire space. Rather, it means that the wave speed profile could be inhomogeneous and/or anisotropic, but the operator learned is for that fixed profile c(x). Hence, to learn the parameter-independent operator Q, the input function f as initial conditions would consist of $u(x,\;T^{train})$ that has prior knowledge of the c(x) profile. During the training stage, for a given f, $u(x,\;T^{predict})$ needs to be provided. After learning this nonparametric operator, Q (for this fixed of c(x)) would become f-independent or the initial-condition-independent operator. Q would be ready to predict the wavefield $u(x,\;T^{predict})$ with any other arbitrary initial conditions (f) as input. In this case, the boundary conditions can also be fixed, irrespective of being Dirichlet, Neuman, or mixed boundary conditions.

Alternatively, if the problem is parameterized and the entire displacement wavefield u(x,t) in a material or structure (i.e., at every spatial point (x,y,z)) over a period (0−T) is asked to find any arbitrary wave speed profile of c(x), then it is necessary to have several random wave speed profiles to train the parametric operator Q. Here, in this setup, to learn the parametric neural operator Q, the input function f would consist of c(x), and the output function would consist of $u(x,\;t_{0}-T)$. Several such random input functions and their corresponding u(x,t) should be used for the training. After learning, for any given c(x) profile (different from what was used in training), the parametric Q would predict the full wavefield u(x,t).

DeepONet and FNOs are both applicable for parametric and nonparametric PDEs. However, their strength lies in mapping and learning the parametric PDEs. Generally speaking, any physical system governed by PDEs can be expressed as an integral operator mapping from one function to another function through a kernel function or a Green’s function.

### 2.2. DeepONet

DeepONet, proposed by Lu et al. [79] is one of the most widely adopted neural operator frameworks. Following the problem statement in Section 2.1, DeepONet approximates the solution operator Q:S→U, where S is the space of input functions such as initial displacement $u_{0}(x)$ or initial velocity $v_{0}\left(x\right)$, and U is the solution space containing the corresponding wavefield u(x,t).

To learn the mapping, DeepONet uses a two-branch architecture. The **branch network** takes as input a discretized form of the function v(x) (e.g., the set of initial velocity $v_{0}\left(x\right)\;$or initial displacement at $u_{0}(x)$ sensor points), while the **trunk network** takes spatiotemporal coordinates (x, y, z, t) as input. Outputs from the two networks are then combined by the inner product to obtain the final prediction. Equation (7) represents the final prediction.

(7) $$Q\left(S\right)(x,\;t)=\sum_{k=1}^{q}B_{k}(v(x)).T_{k}(x,t)$$

Here, $B_{k}(v(x))$ and $T_{k}\left(x,t\right)\;$denote the outputs of the branch and trunk networks, respectively, and q is the latent dimension. This architecture allows the network to learn a generalized operator that can predict solutions at arbitrary spatiotemporal coordinates for a wide range of initial and boundary conditions. The final prediction is compared to the actual wave displacement field to calculate the loss, which is minimized through traditional backpropagation methods [86] to optimize the weights and biases.

**Figure 5:**
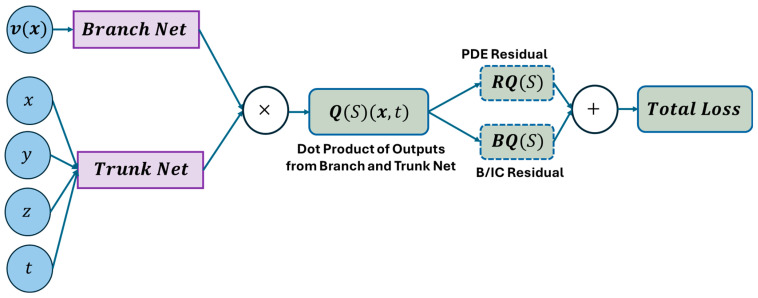
A schematic architecture of DeepONet (physics-informed variant). Reproduced from [91].

There are a few variants of DeepONet, namely physics-informed DeepONet [85,87,88], multiple-input DeepONet [89,90] and many more. Among these variants of DeepONet, only physics-informed DeepONet has been utilized to simulate wave propagation. Figure 5 represents the architecture of a physics-informed DeepONet to model wave propagation. The architecture of the model follows the exact method explained in this section instead of the only weak formation of the loss function to incorporate the physics in the model. The detailed loss calculation formulation can be found here [91]. The pseudocode presented in Algorithm 1 gives readers a clear concept of DeepONet to simulate wave propagation.
**Algorithm 1** DeepONet $\mathrm{Require}:\;\mathrm{Dataset}\;{\left[\left(v^{\left(i\right)}(x),x^{\left(i\right)}\right),u^{\left(i\right)}\right]}_{i=1}^{N}$                                                                               ▹x=(x, y, z, t)$\mathrm{Require}:\;\mathrm{Branch}\;\mathrm{Net}\;B:R^{m}\to R^{q},\;\mathrm{Trunk}\;\mathrm{Net}\;T:R^{4}\to R^{q}\;\;\;\;\;\;\;\;\;\;\;\;\;\;\;\;\;\;\;\;\;\;\;\;\;\;\;\;\;\;\;\;\;\;\;\;▹m=\mathrm{dimension}\;\mathrm{of}\;v^{\left(i\right)}$
1: $\mathrm{Initialize}\;\mathrm{weights}\;\theta_{b},\theta_{\tau }$
2: for epoch=1 to E do
3:              for i=1 to N do 
4:                          $B^{\left(i\right)}\leftarrow B\left(v^{\left(i\right)};\theta_{b}\right)\;\;\;\;\;\;\;\;\;\;\;\;\;\;\;\;\;\;\;\;\;\;\;\;\;\;\;\;\;\;\;\;\;\;\;\;\;\;\;\;\;\;\;\;\;\;\;\;\;\;\;\;\;\;\;\;\;\;\;\;▹\;\mathrm{Branch}\;\mathrm{input}\;\mathrm{from}\;\mathrm{discretized}\;v(x)$
5:                          $T^{\left(i\right)}\leftarrow T\left(x^{\left(i\right)};\theta_{\tau }\right)\;\;\;\;\;\;\;\;\;\;\;\;\;\;\;\;\;\;\;\;\;\;\;\;\;\;\;\;\;\;\;\;\;\;\;\;\;\;\;\;\;\;\;\;\;\;\;\;\;\;\;\;\;\;\;\;\;\;\;\;\;\;\;\;\;\;\;\;\;\;\;\;\;\;\;▹\;\;\mathrm{Trunk}\;\mathrm{input}:\;x=(x,y,z,t)$
6:                          ${\hat{u}}^{\left(i\right)}\leftarrow \sum_{k=1}^{q}\;\;B_{k}^{\left(i\right)}\cdot T_{k}^{\left(i\right)}\;\;\;\;\;\;\;\;\;\;\;\;\;\;\;\;\;\;\;\;\;\;\;\;\;\;\;\;\;\;\;\;\;\;\;\;\;\;\;\;\;\;\;\;\;\;\;\;\;\;\;\;\;\;\;\;\;\;\;\;\;\;\;\;\;\;\;\;\;\;\;\;\;\;\;\;\;\;\;▹\;\;\mathrm{Operator}\;\mathrm{prediction}\;$
7:                          if Physics-informed then
8:                                   $R_{Q}^{\left(i\right)}\leftarrow \;\mathrm{PDE}\;\mathrm{residual}\;\mathrm{at}\;x^{\left(i\right)}$
9:                                   $B_{Q}^{\left(i\right)}\leftarrow B/\;\mathrm{IC}\;\mathrm{residual}\;\mathrm{at}\;x^{\left(i\right)}$
10:                             $L\leftarrow \lambda_{1}{\left\|R_{Q}^{\left(i\right)}\right\|}^{2}+\lambda_{2}{\left\|B_{Q}^{\left(i\right)}\right\|}^{2}$
11:                   **else**
12:                             $L\leftarrow MSE\left({\hat{u}}^{\left(i\right)},u^{\left(i\right)}\right)$
13:                    **end if**
14:                    $\mathrm{Update}\;\theta_{b},\theta_{\tau }\;\mathrm{via}\;\mathrm{backprop}\;\mathrm{on}\;L$
15:            **end for**
16: **end for**


### 2.3. Physical Understanding of DeepONet

Sometimes, it is confusing and challenging to recognize why and how DeepONet could have the potential to understand the physics of a system. In relation to elastic and acoustic wave propagation, the concept of DeepONet is explained herein. Here, it is necessary to reiterate the eigenfunction expansion method for solving the partial differential equation (PDE) from the fundamental. It is known that any solution to the governing PDE would be the superposition of dominant eigenfunctions multiplied by their respective contribution coefficients. The solution of the wave PDE in Equation (1) could be written as

(8) $$u\left(x,t\right)=\sum_{i=0}^{M}a_{i}(t)\phi_{i}(x)$$

where $\phi_{i}(x)$ is the i-th basis or space-dependent eigenfunction and $a_{i}(t)$ is the i-th temporal eigenfunction associated with its respective participation coefficient. M is the number of modes or basis or eigenfunctions considered in the superposition. Separating the participation coefficients and expressing the temporal and spatial functions into one function, the displacement wavefield could visualize

(9) $$u\left(x,t\right)=\sum_{i=0}^{M}C_{i}\varphi_{i}(x,t)$$

Specific to the problem presented in Equation (1), if the initial conditions $u_{0}\left(x\right)\;$ and $v_{0}\left(x\right)\;$ are known and a wavefield is computationally or experimentally found at some spatiotemporal space (x,t) or at some sensor locations, then these known datasets create an opportunity to find the internal mapping functions that consist of inherent eigenfunctions of the system. The objective of the problem is to solve the PDE and find the displacement wavefield at any point in space (x) and at any time (t). Hence, according to Equation (9), if somehow the participation coefficients $C_{i}$ and the eigenfunctions $\varphi_{i}(x,t)$ are found, then the solution at any point in space (x) and at any time t could be explicitly obtained. With this fundamental background, DeepONet shoots for finding the solutions for $C_{i}$ and $\varphi_{i}(x,t)$ for M number of modes. By now, it is clear that q (latent dimension) in Equation (7) is equivalent to the parameter M that indicates how many eigenfunctions are to be considered in the solution and is taken as an input from the user. Branch net takes the initial conditions $B_{k}(v(x))$ and tries to find the $C_{i}$ coefficients for all q≡M modes. Branch net tries to answer the contribution of each mode or each eigenfunction to the final solution based on the initial condition. Whereas trunk net $T_{k}(x,t)$ in Equation (6) tries to find the eigenfunction $\varphi_{i}(x,t)$ for all q≡M modes. Trunk net tries to evaluate the response of each mode at each point in space and time (x,t).

Having these two answers, based on Equation (9), it is obvious that the output from the branch net and trunk net needs to be multiplied to find the displacement wavefield $u\left(x,t\right)$. However, in order to train the model, the total loss is minimized based on the initial condition and the user-provided wavefield as training data. Once the model is trained, it is expected to find the displacement wavefield $u\left(x,t\right)$ at any query point $\left(x,t\right)$, given any initial conditions $u_{0}\left(x\right)\;$ and $v_{0}\left(x\right)$. Please note, here, the initial conditions are not required to be the same as the initial conditions used for training. This philosophy is the heart of DeepONet and is much faster and more generalized than PINNs.

**Figure 6:**
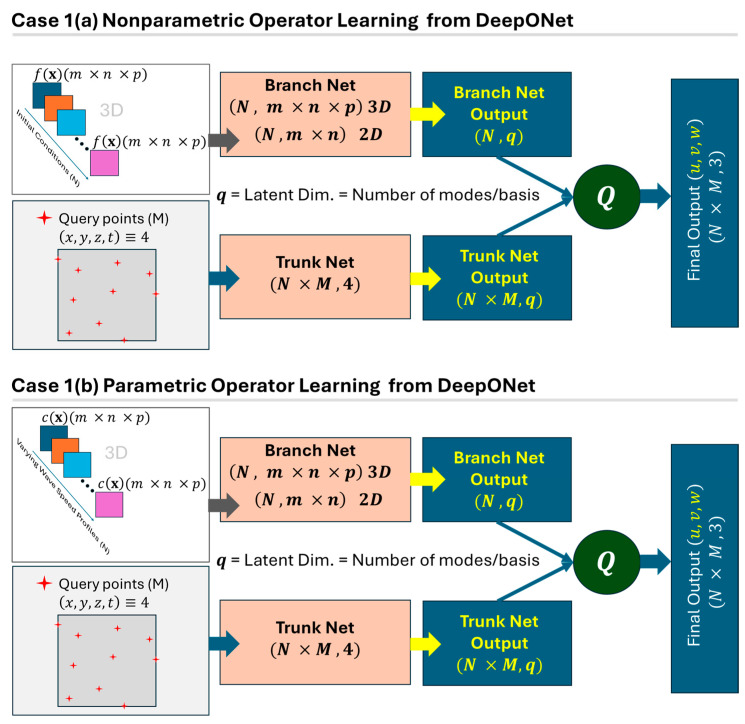
A schematic architecture of two cases for nonparametric and parametric PDE modeling using DeepONet (physics-informed variant).

Now, with the above concepts, generally speaking, DeepONet does not explicitly construct the kernel function in Equation (6) but represents the operator via latent basis functions and coefficients. Comparing Equations (6) and (9), it can be said that DeepONet replaces the continuous effect of the kernel integral with a weighted combination of learned basis functions, where the weights depend on the input function, and the basis functions capture how each point in the domain responds. Figure 6 shows two different cases with parametric and nonparametric PDEs modeled using DeepONet for wave propagation.

### 2.4. Fourier Neural Operator (FNO)

In 2020, Li et al. [80] introduced the FNO. In contrast to DeepONet, FNO uses the convolutional operator in Fourier space instead of the kernel integral operator. These modifications allow the model to capture long-range dependencies more efficiently and to generalize across varying discretization. Unlike DeepONet, FNO avoids explicit basis construction, and it implicitly learns the operator kernel by manipulating Fourier modes.

In the context of the 3D wave propagation problem described in Section 2.1, the goal is to approximate the operator Q that maps the input function v(x) to the wavefield u(x). For nonparametric PDEs, the input function v(x) can refer to the initial displacement $u_{0}(x)$ or initial velocity $v_{0}(x)$ at time zero or at any other time t=τ, labeling ($u_{0}\left(x,\tau \right))\;and\;v_{0}(x,\tau )$, respectively, or any combinations of these functions (e.g., f(x)). It is important to note that, in many wave propagation problems, the initial conditions at t=0 do not contain essential features, or sometimes the displacement and velocity values are zero. Thus, instead of training with the data at t=0, it is essential to provide the data at t=τ to learn physics. This is considered a forward nonparametric problem under FNO. Alternatively, a forward parametric problem could be better suited for FNO, where a spatially varying wave speed c(x) could be input to an FNO model and displacement or velocity wavefields are output from the model. The power of FNO comes from learning global, resolution-independent mappings between functions in Fourier space—whether those functions encode variable parameters (as in parametric PDEs) or time-evolving fields (as in nonparametric cases).

**Figure 7:**
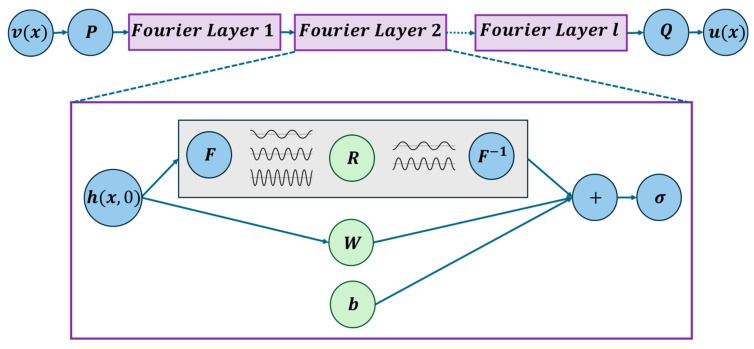
A schematic architecture of FNO. Reproduced from [80].

Irrespective of input types, the input is first lifted to a high-dimensional latent space using a lifting operator P, which can be a shallow NN or just a linear transformation, resulting in h (x, 0)=P(f(x)). Where the second argument of the function h (x, j) signifies the j-th Fourier layer. This h(x,j) is passed through a sequence of Fourier layers. The h (x, 0) is fed to the first Fourier layer, and the process is subsequently repeated. The operation at the jth layer is given by

(10) $$h(x,j+1)=\sigma \left(W_{j}h(x,j)+F^{-1}\left[R_{j}\cdot F[h(\cdot ,j)]\right](x)+b_{j}\right)$$

where F and $F^{-1}$ are the Fourier and inverse Fourier transforms, $R_{j}$ is a learnable parameter in Fourier space, $W_{j}$ is a pointwise linear operator in physical space, and $b_{j}$ is a bias term. The nonlinearity σ(⋅), typically a GELU [92] or ReLU [93] function or any activation function, adds expressiveness to the model. After passing through all L layers, the final representation h(x,L) is projected back to the output space by a decoder Q, yielding the predicted wavefield $u_{\theta }(x,t)=Q(h(x,L))$. Later, the loss calculation is carried out in the traditional way, or sometimes, based on the problem, physics inclusion is carried out in the weak form. Figure 7 provides a schematic representation of the FNO model. Algorithm 2 presents the pseudocode for the FNO applied to solving the wave equation.
**Algorithm 2** FNO$\mathrm{Require}:\;\mathrm{Dataset}\;{\left\{\left(v^{\left(i\right)}\left(x\right),u^{\left(i\right)}(x)\right)\right\}}_{i=1}^{N}$$\mathrm{Require}:\;\mathrm{Lifting}\;\mathrm{operator}\;P:R^{m}\to R^{d},\;\mathrm{Fourier}\;\mathrm{layers}\;F_{j}\;\mathrm{for}\;j=1,\ldots ,L,\;\mathrm{decoder}\;Q$1: $\mathrm{Initialize}\;\mathrm{weights}\;\theta_{P},{\left\{W_{j},R_{j},b_{j}\right\}}_{j=1}^{L},\theta_{Q}$
2: for epoch=1 to E do 
3:            for i=1 to N do 
4:                         $h_{0}^{\left(i\right)}(x)\leftarrow P\left(v^{\left(i\right)}(x)\right)\;\;\;\;\;\;\;\;\;\;\;\;\;\;\;\;\;\;\;\;\;\;\;\;\;\;\;\;\;\;\;\;\;\;\;\;\;\;\;\;\;\;\;\;\;\;\;\;\;\;\;\;\;\;\;\;\;\;\;\;\;\;\;\;\;\;\;\;\;\;\;\;\;\;\;\;\;\;▹\;\mathrm{Lift}\;\mathrm{to}\;\mathrm{latent}\;\mathrm{space}\;$
5:                         for j=0 to L−1 do 
6:                                    ${\hat{h}}_{j+1}^{\left(i\right)}(x)\leftarrow F^{-1}\left[R_{j}\cdot F\left[h_{j}^{\left(i\right)}\right]\right](x)\;\;\;\;\;\;\;\;\;\;\;\;\;\;\;\;\;\;\;\;\;\;▹\;\mathrm{Fourier}\;\mathrm{transform}\;\mathrm{and}\;\mathrm{filtering}$
7:                                    $h_{j+1}^{\left(i\right)}(x)\leftarrow \sigma \left(W_{j}h_{j}^{\left(i\right)}(x)+{\hat{h}}_{j+1}^{\left(i\right)}(x)+b_{j}\right)\;\;\;\;\;\;\;\;\;\;\;\;\;\;\;\;\;\;\;\;\;\;\;\;\;\;\;\;\;▹\;\mathrm{Nonlinear}\;\mathrm{activation}$
8:                         **end for**
9:                         ${\hat{u}}^{\left(i\right)}(x)\leftarrow Q\left(h_{L}^{\left(i\right)}(x)\right)\;\;\;\;\;\;\;\;\;\;\;\;\;\;\;\;\;\;\;\;\;\;\;\;\;\;\;\;\;\;\;\;\;\;\;\;\;\;\;\;\;\;\;\;\;\;\;\;\;\;\;\;\;\;\;\;\;\;\;\;\;\;\;\;\;\;\;▹\;\mathrm{Decode}\;\mathrm{output}\;\mathrm{wavefield}\;$
10:                   $L\leftarrow MSE\left({\hat{u}}^{\left(i\right)},u^{\left(i\right)}\right)$
11:              Update all parameters via backprop on L
12:             **end for**
13: **end for**

### 2.5. Physical Understanding of FNOs

Even it is more confusing and challenging than DeepONet to recognize why and how FNOs can be faster and have broader potential for understanding the physics of a system. The concept of FNOs is explained herein in relation to elastic and acoustic wave propagation. FNOs being mesh independent, a small set of low-resolution data can predict a solution with improved finer resolution while the spatial dimension of the problem remains the same. FNOs have additional potential to solve inverse problems. As discussed under general neural operator, an operator Q maps from one function to another function. Hence, there is no harm if one can place multiple such operators in a sequence one after another (refer l layers in FNOs). Every operator performs the same action as presented in Equation (6) and needs their respective kernel function for their respective input–output mapping. Knowing how DeepONet performs the mapping from Section 2.3, it can be said that the FNO performs the mapping differently than its predecessor. The FNO explicitly approximates the integral operator in Equation (6) using Fourier transforms using the convolution theorem as follows:

(11) $$Q\left(f\right)\left(x,t\right)=\int_{\Omega }\;K(x-\xi ,\;t;\tau )f(\xi ,\tau )d\xi$$

The reason behind this thinking is that the influence of the respective input function f(ξ,τ) on the respective output function u(x,t) depends only on the relative distance between x and ξ, not on their absolute positions. As convolution in physical space is equivalent to elementwise multiplication in Fourier space, the Fourier transform of Equation (11) can be read as follows:

(12) $$F\left[Q\left(f\right)\left(x,t\right)\right]=F\left[K(x-\xi ,\;t;\tau )\right]\cdot F\left[f(\xi ,\tau )\right]\;$$

As the kernel function is not known, its Fourier coefficients are also not known; thus, the Fourier coefficients of the kernel function can be the learning parameter replaced by R as depicted in Figure 7. After taking the inverse Fourier transform, the mapped function can be retrieved as follows:

(13) $$Q\left(f\right)\left(x,t\right)=F^{-1}\left[R\;\cdot F\left[f(\xi ,\tau )\right]\right]$$

Based on this explanation, now it is easier to understand Equation (10), where there are multiple FNO layers present, designated with index j. Every FNO has its own inputs and respective outputs in a sequence. $W_{j}$, the pointwise linear operator, $b_{j}$, the bias and activation function σ(·) in Equation (10) are now self-explanatory from the fundamentals of neural networks.

Further, it is useful to visualize the difference between DeepONet and FNO. A comparative analysis is presented in Table 1.

### 2.6. Application Cases of FNO

Here, two cases that are widely occurring in NDE/SHM that could be benefited by FNO are explained from the fundamental physics and mathematical perspectives. These two cases are listed as (a) Case 1: Forward problem and (b) Case 2: Inverse problem. At its heart, the FNO is a data-driven approximation of a nonlinear operator mapping one function space to another. In physical systems like wave propagation, the governing PDE (like the elastic wave equation) describes how a wavefield evolves over space and time based on material properties like wave speed.

Case 1(a): Predicting the displacement wavefield at a later time with a given displacement wavefield at an earlier time with a known and fixed velocity profile (for nonparametric operators) in the material. Velocity and density could be homogeneous or nonhomogeneous, isotropic or anisotropic throughout the material. This will not be relevant when one is interested in finding the wavefield for different random velocity or density profiles.

(14) $$Q\;:u_{0}\left(x,T^{train}\right)\;|\;\;v_{0}\left(x,T^{train}\right)\;\overset{yields}{\to }\;\;\;u(x,T^{predict})$$

**Figure 8:**
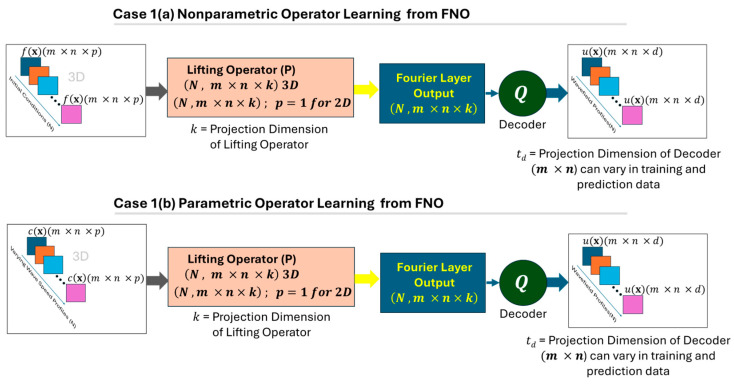
A schematic architecture of two cases for nonparametric and parametric PDE modeling using FNO.

Case 1(b): Predicting the displacement wavefield u(x,t) with a given velocity or density profile (considered parameters in wave propagation). The training will consist of a velocity or density map c(x), which could be homogeneous or nonhomogeneous, isotropic or anisotropic throughout the material, and several random states should be trained. This will be a mandatory mapping process when one is interested in finding the wavefield for different random velocity or density profiles. Figure 8 shows two different cases with parametric and nonparametric PDEs for modeling wave propagation with the FNO.

(15) $$Q\;:c\left(x\right)\;\overset{yields}{\to }\;\;\;u(x,t)$$

Case 2: Inferring the material velocity profile from observed wave propagation or recorded wavefield data from specific sensor points. In this case the velocity at each pixel located at x is considered to have a different velocity and can be expressed as a function c(x).

(16) $$Q^{I}\;:u(x,t)\;\overset{yields}{\to }\;\;\;c(x)$$

The core idea is to approximate the operators (Q or $Q^{I}$) not in the physical space, but in the Fourier space, where global spatial dependencies (like wave propagation, reflection, and dispersion) are naturally captured through Fourier modes. It is known that Fourier transforms a wavefield into a set of standing and traveling wave modes or sinusoids. The FNO learns how the amplitude and phase of this mode should evolve, depending on the underlying material properties and source characteristics. This was achieved through finding the complex multiplier or the weight tensor $R_{j}\;$per mode that are learned through the training process. Next, by transforming the learned updates back to physical space, the FNO reconstructs the updated wavefield or predicts material properties.

In the forward problem (Case 1), it predicts how a wavefield will propagate over time based on its current state, essentially learning a surrogate model for time-stepping. 

In the inverse problem (Case 2), the FNO effectively learns to invert the wave propagation operator by associating observed wavefield patterns with their generating velocity profiles.

## 3. NO Applications in Wave Propagation

### 3.1. Wave Propagation with DeepONet

DeepONet has been widely adopted in several research areas. However, the literature lacks in quantity when it comes to simulating wave propagation. Only a few research works in 2024 have been found. Notably, most of these works extended the standard DeepONet architecture to improve performance in solving wave equations.

**Figure 9:**
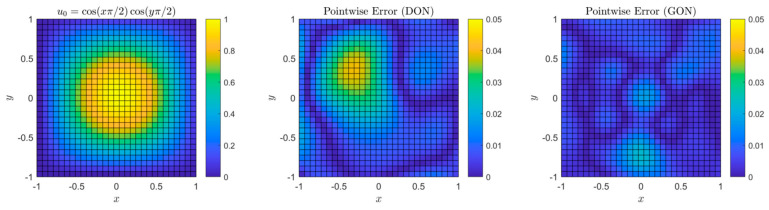
(**Left**) initial condition; (**middle**) pointwise error at *t* = 1.5 using DeepONets; (**right**) pointwise error at *t* = 1.5 using GreenONets, adapted from [91].

Aldirany et al. [91] first introduced **GreenONets**, a variant of DeepONet inspired by Green’s functions. The model’s performance was evaluated using the ground truth and vanilla DeepONet while solving the linear wave equation in the 1D and 2D homogeneous and heterogeneous domains. Although GreenONet demonstrated improved accuracy in these cases, the authors raised concerns about the generalizability of the model. A comparative analysis of the performance of DeepONet and GreenONet is presented in Figure 9.

Later, Zhu et al. [94] proposed another variation of the DeepONet, named **Fourier DeepONet**. The authors combined the concepts of FNOs and DeepONet to perform full waveform inversion (FWI). While the original DeepONet architecture was largely retained, the final dot product of the branch and trunk networks was passed through one Fourier layer followed by three U-Fourier layers to enhance the model’s ability to capture high-frequency components. The inclusion of U-Fourier layers resulted in improved predictive accuracy but also introduced a higher computational cost, as U-Fourier layers [95] are more expensive to train. The proposed model demonstrated robust performance across a range of datasets, including flat layers, curved layers, faulted media, and style-transferred geological configurations.

In another study, Guo et al. [96] introduced Inversion DeepONet, a DeepONet-based architecture incorporating an encoder–decoder framework. This approach effectively reduced the dependence on large training datasets by using only source parameters, such as frequency and location, as input. It also addressed the limitations associated with the standard dot product operation between the trunk and branch networks, which often leads to suboptimal performance. The literature suggests that a substantial portion of DeepONet-related work is concentrated in geophysical applications. While most studies propose architectural variations of DeepONet, Li et al. [97] focused on developing more generalized datasets to overcome the limitations of the existing OpenFWI data. As a result, they introduced GlobalTomo, a comprehensive dataset encompassing 3D acoustic and elastic wave propagation for full waveform inversion and seismic wavefield modeling.

**Figure 10:**
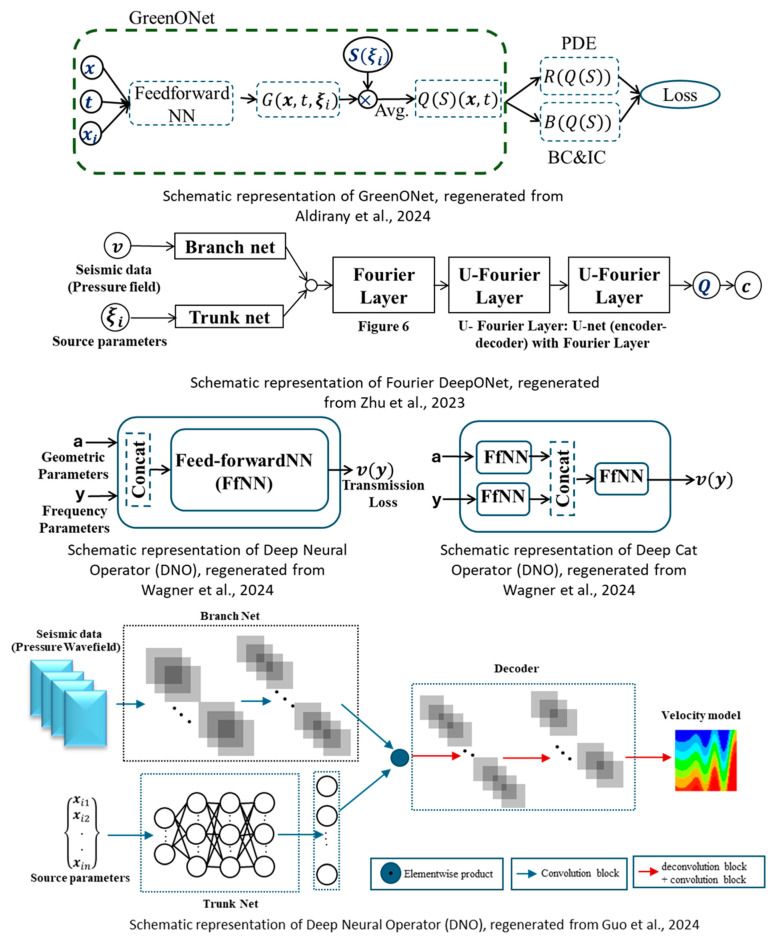
Different variants of DeepONet architectures to model wave propagation [91,94,96,98].

To date, only a single research article has been found utilizing the concept of DeepONet in the field of material property characterization. Wagner et al. [98] explored four different operator learning approaches to investigate the material properties of sonic crystals by solving the acoustic wave equation. Among these, two are DeepONet and FNO, whereas **deep neural operator (DNO)** and **deep cat operator (DCO)** are the two new ones, inspired by the concept of the vanilla DeepONet. These two approaches outperformed DeepONet and FNO. However, the study did not compare the results with any simulations generated with the traditional models, which questions the accuracy of the performance of these four models. Figure 10 depicts the architecture of the different modifications of DeepONet, providing a clear idea of each of the model’s algorithms. Table 2 represents a summary of all the research work discussed in this section.

### 3.2. Wave Propagation with FNOs

This section provides a comprehensive review of recent studies employing FNOs for solving wave equations in applied domains. The integration of physics-based wave modeling with data-driven approaches has gained momentum across diverse disciplines, including structural health monitoring [99,100], material design [101,102], and medical imaging [103]. Notably, geophysics stands out for adapting NOs. Currently, scholars in this field are actively harnessing this new approach to reconstructing higher-resolution earth models.

Yang et al. [104] used the NO for the first time in 2021 to simulate the seismic wavefield. To train the FNO, 5000 random velocity models were generated using the spectral element method (SEM). To create a varied range of velocity models, the authors used the von Karman covariance function and different combinations of source receivers and their locations. The trained model successfully reconstructed smooth heterogeneous surfaces and surfaces with sharp discontinuities and shorter wavelengths. This approach bypasses the need for adjoint wavefields, which are typically required in numerical methods for full waveform inversion (FWI). The study concludes that a larger training dataset improves the model’s ability to generalize. It also enables more accurate capture of complex wave phenomena such as reflections, which are challenging for FNOs when trained on limited data.

**Figure 11:**
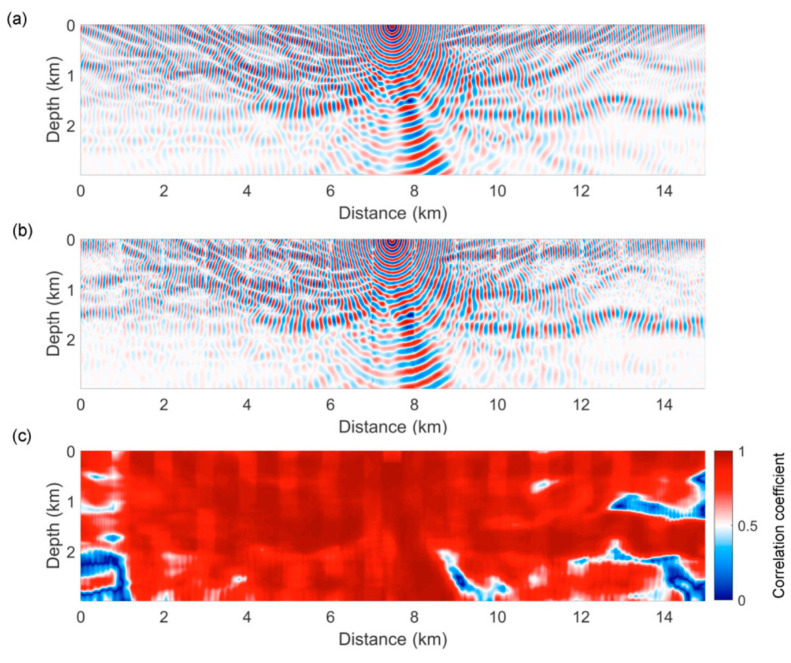
Wavefields at 13 Hz from (**a**) the finite-difference method, (**b**) from the FNO, and (**c**) the correlation coefficients between (**a**,**b**) corresponding to the original Marmousi model [105].

While the previous research group focused on time domain FWI, the very next year, Song and Yang [105] trained FNOs on frequency domain wavefield extrapolation. The research group specifically focused on mapping the low-frequency wavefield (5–12 Hz) to a higher-frequency wavefield (13–30 Hz). Synthetic data were generated using the FDM based on the Marmousi model to train the FNO. The focus of this study was to obtain dispersion-free high-frequency wavefields for large-scale subsurface models with less computational cost. The trained model was experimented with three test cases: a strongly smoothed model, a moderately smoothed model, and the original Marmousi model. For the first two cases, the results from both FDM and FNOs were in good agreement. However, for the original Marmousi model, the correlation coefficient was comparatively high, specifically in the shallow low-frequency region where FDM suffered from higher dispersion error. Figure 11 shows the performance comparison of the original Marmousi model. The FNO proved its worth in both surpassing the FDM in dispersion error and computational cost. According to this study, the trained FNO model was two orders of magnitude faster than FDM.

**Figure 12:**
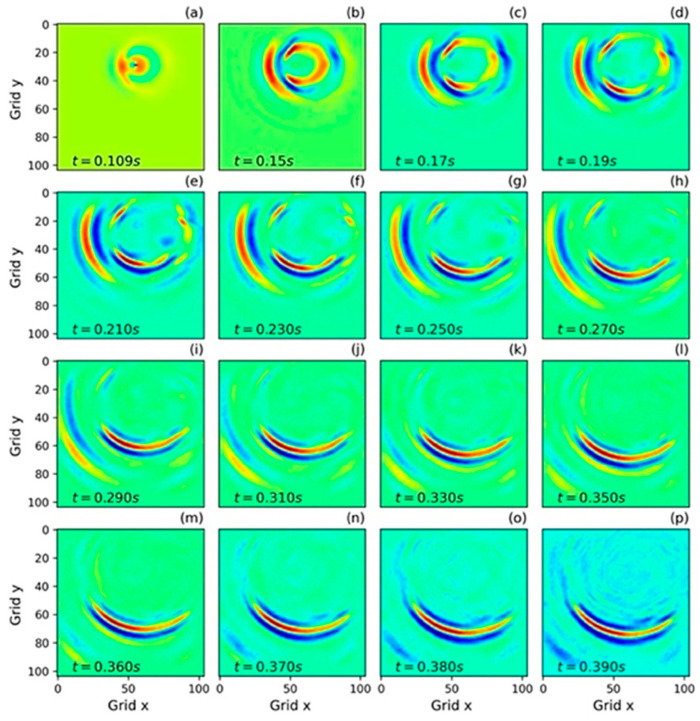
Nine snapshots of the x component of the wavefields generated with the FNO. With dimension projection width 60 and 33 Fourier models, adapted from [106].

**Figure 13:**
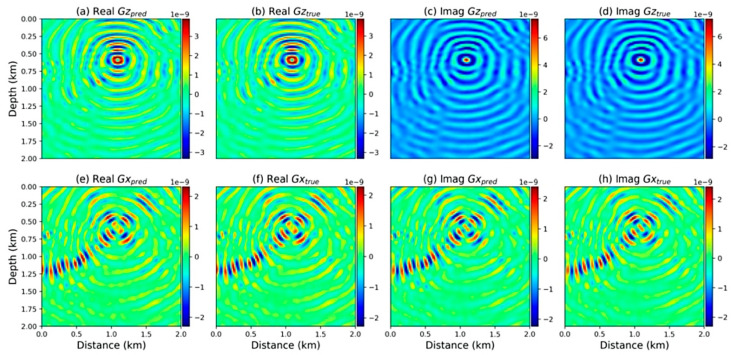
The training results in the frequency domain. The main frequency is 31 Hz, and the source location is located at 0.25 km in depth and 1.37 km in distance from the model. (**a**,**b**) are the real parts of the wavefields in the z direction. (**c**,**d**) are the wavefields in the x direction. (**e**,**f**) are the real parts of the fields in the x direction. (**g**,**h**) are the imaginary parts of the fields in the x direction, adapted from [106].

For the first time, Zhang et al. [106] investigated the incorporation of NOs for 2D elastic wave propagation in both time and frequency domains in 2022. The time domain datasets were trained using 100 velocity models with varying shapes and source locations. To generate the frequency domain training dataset, 800 models were derived from a 3D overthrust model of varying source locations and frequencies within a specific range. The trained FNO performed well in both cases, as shown in Figure 12 and Figure 13. However, the performance of the model degrades noticeably in the time domain case with increasing time with amplitude mismatch and source distortion effect. In the frequency domain, the model faced challenges in simulating high-frequency components with intricate patterns.

From 2021 to 2022, a small number of research works incorporated NOs for simulating acoustic and elastic wave equations. A recurring trend in the literature is the use of traditional numerical methods to generate training datasets for specific model configurations. While training the FNO, the overarching structure of the model remains consistent, but key hyperparameters are tuned according to the specific problems being addressed. However, 2023 marked a significant diversification in model architectures and solution domains. There were also notable advancements in robust generalization techniques and a growing shift toward application-focused research beyond geophysics into other fields. Li et al. [107] first introduced a variation of the FNO model, **parallel FNO (PFNO)**, to predict the 2D acoustic wave equation to increase the generalization capability of the model. Instead of using one velocity model with variations, the proposed method trains several velocity models using different FNOs in the same simulation setup. Though the training of PFNO is computationally very demanding, the generalization capability of this model is beyond PINNs and FNOs.

Lehmann et al. [108] first utilized NOs to simulate elastic wave propagation in 3D heterogeneous isotropic materials. The authors proposed another variation of the NO named the **U-shaped neural operator (UNO)**. In this architecture, the Fourier layers are arranged in an encoder–decoder structure, which allows skip connections, offering easier training and balance of capturing global and local features. The whole experiment has been carried out with the layer model, introducing heterogeneities in each layer using Karman random fields. Kong et al. [109] also studied 3D elastic waves, focusing on modeling both P and S waves. The authors compared the predictability of FNO and UNO for low-velocity zones and vertical slab structures. Though UNO outperformed FNO on small-scale simulations, it was slower by a factor of 4.

While most of the research articles demonstrate the fact that FNO faces challenges in capturing the wavefield with evolved time, Middleton et al. [110] experimented with multiple sets of input–output ratios simulating the 2D linear acoustic wave equation in the free field domain to present an overall idea of the model’s capability. Rosofsky et al. [111] proposed PINO, combining the PINN and FNO, including the physics-informed loss term with the FNO structure. The model was tested using the 1D wave equation, the 2D wave equation, and the 2D nonconstant coefficients wave equation. This model faces challenges in the case of higher-magnitude initial data (input value > 1). Thus, as a solution, a normalized wavefield has been sought to train the data. PINO has been successfully applied to simulate the frequency domain acoustic wave equation in vertically transverse isotropic (VTI) media with high accuracy.

**Figure 14:**
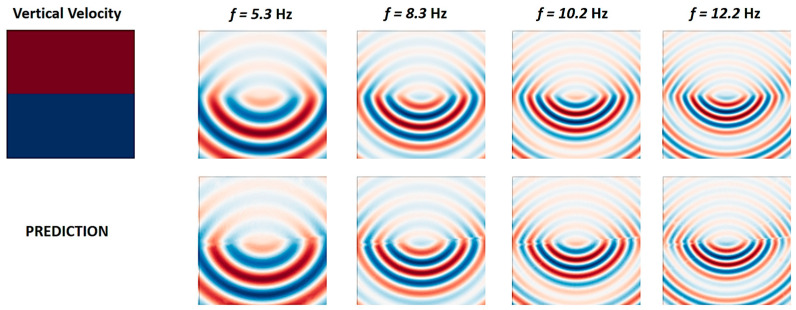
The real component of monochromatic scattered wavefields for the two-layer model obtained using a numerical solver (**top** row) and predicted by the proposed neural network (**bottom** row), adapted from [112].

Konuk and Shragge [112] proposed a novel approach to train PINO without any pre-simulated data. The research group utilized the real and imaginary components of the background wavefield, which were analytically incorporated into the training process. This innovative method allowed the model to accurately mimic wave propagation in anisotropic media. Figure 14 reflects on the predictability of their proposed approach.

Although, currently, NO-based wave propagation study is mostly confined within the geophysics field, Guan et al. [113] first solved a photoacoustic wave equation for simulating photoacoustic tomography (PAT) with FNOs. To address this problem, the researchers modified the architecture of the FNO with an incorporation of the convolutional neural network (CNN) model. Instead of using only the Fourier layer as the linear operator, the authors combined the CNN and Fourier layers. In this context, the CNN has been incorporated to capture the local features from the spatial data (edges, textures, etc.), while the Fourier layer captures the global features (resolution invariance, sharp transition, etc.). The output from the two linear operators is later added and passed to the activation function (GELU). This study experimented with two key hyperparameters: channels and frequency modes. The models have been trained on the breast vascular simulation dataset. The generalization ability of the model has been tested with other models such as breast tumor, Shepp–Logan, synthetic vascular, and Mason—M logo phantoms, where the models performed well in most of the cases. The authors suggested more accuracy by tuning the channels and frequency for robustness. Table 3 summarizes the research works discussed in this section.

## 4. Conclusions

This article underscores the potential and applicability of NOs in wave propagation modeling. Unlike traditional solvers or other SciML methods, NOs directly learn the solution operator of a PDE, enabling rapid and accurate predictions over a distribution of input functions. This review primarily focuses on DeepONet and FNO, along with their different variants. These methods have demonstrated strong generalization and inference efficiency across diverse wave modeling tasks. According to the literature, seismic imaging is comparatively the most active field for successfully incorporating NOs in wave modeling.

The integration of NOs, including FNO, DeepONet, and GreenONet, holds transformative potential for the fields of non-destructive evaluation (NDE) and structural health monitoring (SHM). These frameworks offer a paradigm shift from conventional data-driven models to physics-informed, operator-learning architectures capable of directly learning mappings between infinite-dimensional function spaces. In the context of NDE/SHM, this enables real-time, scalable, and highly generalizable models for tasks such as wavefield reconstruction, damage localization, and forward/inverse problem-solving in complex structures.

Future research directions will likely focus on leveraging FNOs for the efficient modeling of wave propagation phenomena in heterogeneous and anisotropic materials, significantly accelerating simulations used in guided wave-based inspections. DeepONets and GreenONets, with their capacity to learn solution operators for partial differential equations (PDEs), are well-suited for developing surrogate models for inverse problems, such as inferring damage characteristics from sparse measurement data. Additionally, combining these neural operator frameworks with physics-informed loss functions and uncertainty quantification techniques could improve model interpretability and reliability, addressing key challenges in safety-critical SHM applications.

Moreover, these operator-learning models can support the development of digital twins for structural systems, providing near real-time predictive maintenance insights by rapidly simulating the structural response under varying operational and damage scenarios. The synergy between NO architectures and edge computing platforms may further enable onboard, in situ SHM for aerospace, civil infrastructure, and energy sector applications, making advanced NDE/SHM more accessible, adaptive, and autonomous.

Irrespective of several positive outcomes, it is necessary to be aware that the robustness of these models comes at the cost of training larger datasets. Their performance is often sensitive to architectural design, data distribution, and training stability. Future work should explore self-supervised or hybrid training strategies, improved operator expressiveness, and integration of uncertainty quantification to broaden their applicability.

Overall, NOs represent a paradigm shift in scientific computing for wave propagation. Their data efficiency and resolution invariance make them a compelling alternative to mesh-based methods. By bridging computational physics and deep learning, NOs are poised to play a central role in the next generation of simulation tools for wave-based diagnostics and design.

## Figures and Tables

**Table 1:** Comparison of DeepONet and FNO.

Aspects	DeepONet	FNO
Operator type	Approximates via finite basis expansion	Approximates via Fourier-domain convolution
Kernel function	Implicit via trunk net basis and branch net coefficients	Explicit via learned Fourier multipliers
Integral approximation	Discrete latent expansion into number of modes and their contributions $\sum_{i=0}^{M}C_{i}\varphi_{i}(x,t)$	Fourier transform, multiply in spectral space
Global modeling	Through learned basis functions in trunk net	Through Fourier modes capturing global functional behavior
Miscellaneous understanding	Very general; works for arbitrary operators	Most efficient when operator is translation-invariant (convolution-like PDEs)
Parametric PDEs	Yes Input: Parametric function (e.g., wave speed map c(x), initial profile)	Yes Input: Parametric function (e.g., wave speed map c(x), initial profile)
Nonparametric PDEs	Yes Input: Prior field value (e.g., $u(x,T^{train})$)	Yes Input: Prior field value (e.g., $u(x,T^{train})$)
Inverse Problem	No: tough to converge	Yes

**Table 2:** A selective list of studies leveraging DeepONet for modeling wave propagation for addressing different engineering problems.

Authors	Year	Key Objectives	Model Architecture	Type of Wave	Dimension	Type of Medium
Aldirany et al. [91]	2024	Transient wave propagation modeling	DeepONet and GreenONet	Acoustic wave	2D	Homogeneous
Zhu et al. [94]	2024	Full waveform inversion with noise-robust generalization	Fourier DeepONet	Acoustic wave	2D	Heterogeneous
Guo et al. [96]	2024	Improve generalization across source locations and frequencies	Inversion DeepONet	Acoustic wave	2D	Heterogeneous
Li et al. [97]	2024	Accelerated global seismic forward modeling and inversion	DeepONet, Physics-Informed DeepONet	Acoustic wave and Elastic wave	3D	Heterogeneous
Wagner et al. [98]	2023	Fast surrogate modeling of transmission loss in sonic crystals	DeepONet	Acoustic wave	2D	Homogeneous

**Table 3:** A selective list of studies leveraging FNOs for modeling wave propagation for addressing different engineering problems.

Authors	Year	Key Objectives	Model Architecture	Type of Wave	Dimension	Type of Medium
Yang et al. [104]	2021	Fast inference of 2D seismic wavefields across varying source and velocity	Vanilla FNO	Acoustic wave	2D	Heterogeneous
Song and Yang [105]	2022	Predicting high-frequency wavefields from low-frequency inputs	Vanilla FNO	Acoustic wave	2D	Heterogeneous
Zhang et al. [106]	2022	Time extrapolation of wavefields for seismic analysis	Vanilla FNO	Elastic wave	2D	Heterogeneous
Li et al. [107]	2023	Forward modeling across diverse velocity models for full waveform inversion	Parallel FNO (PFNO)	Acoustic wave	2D	Heterogeneous
Lehmann et al. [108]	2023	Simulating 3D elastic ground motion for earthquake hazard assessment	U-Shaped FNO (UNO)	Elastic wave	3D	Heterogeneous
Kong et al. [109]	2023	Real-time simulation of 3D ground motion for subsurface imaging and seismic inversion	UNO and Vanilla FNO	Elastic wave	3D	Homogeneous
Middleton et al. [110]	2023	Learning long-term acoustic wave propagation from short input in a free-field simulation	Tensorized FNO (TFNO)	Acoustic wave	2D	Homogeneous
Rosofsky et al. [111]	2023	Surrogate modeling of wave equation	Physics-Informed FNO (PIFNO)	Elastic wave	1D, 2D	Homogeneous
Konuk and Shragge [112]	2023	Generalizing frequency domain AWE solutions for anisotropic media across frequencies	PIFNO	Acoustic wave	2D	Anisotropic VTI
Guan et al. [113]	2023	Fast modeling of broadband photoacoustic wave propagation for image reconstruction applications	Vanilla FNO	Acoustic waves	2D	Homogeneous
